# Environmental context reveals distinct regulatory patterns in field-grown versus in vitro symbiotic protocorms of *Dactylorhiza majalis* (Orchidaceae)

**DOI:** 10.1186/s12870-026-09554-y

**Published:** 2026-07-20

**Authors:** Julita Minasiewicz, Marcin Jąkalski, Marie-Laure Martin, Alexandra Launay-Avon, Tomáš Figura, Piotr Mleczko, Monika M. Lipińska, Konrad Ślusarz, Marc-André Selosse, Etienne Delannoy

**Affiliations:** 1 Department of Plant Taxonomy and Nature Conservation, Faculty of Biology, University of Gdańsk, ul. Wita Stwosza 59, Gdańsk, 80-308 Poland; 2 Center for Applied Genomics and Bioinformatics, University of Gdańsk, ul. Wita Stwosza 59, Gdańsk, 80-308 Poland; 3 Université Paris-Saclay, CNRS, INRAE, Université Evry, Université Paris Cité Institute of Plant Sciences Paris-Saclay (IPS2), Gif sur Yvette, 91190 France; 4 INRAE, AgroParisTech, UMR MIA-Paris, Université Paris-Saclay, Paris, France; 5 Institute of Botany ASCR, Department of Mycorrhizal Symbioses, Lesní 322, Průhonice, 252 43 Czech Republic; 6 Department of Experimental Plant Biology, Faculty of Science, Charles University, Viničná 5, Prague, 12844 Czech Republic; 7 Faculty of Biology, Institute of Botany, Jagiellonian University, ul. Gronostajowa 3, Kraków, PL-30-387 Poland; 8 Centro de Investigación Jardín Botánico Lankester, Universidad de Costa Rica, Cartago, Costa Rica; 9 Institut de Systématique, UMR 7205 – CNRS, MNHN, UPMC, EPHE), Muséum national d’Histoire naturelle, Sorbonne Universités, 57 rue Cuvier, Évolution, Biodiversité, Paris, 75005 France

**Keywords:** Mycorrhiza, Symbiosis, Germination, Environment, Orchidaceae, Transcriptome, RNA-Seq, Gene Expression Regulation, Plant / physiology, Membrane Transport Proteins/metabolism

## Abstract

**Background:**

Understanding orchid mycorrhizas and symbiotic germination has long depended on in vitro models, yet these systems fail to capture the environmental forces shaping natural symbiosis. Here, we use RNA-seq to compare symbiotic and asymbiotic protocorms (the post-embryonic organ characteristic of orchid germination) grown in vitro with those developing in the field, allowing us to resolve how ecological context reshapes early plant physiology and plant-fungus interactions.

**Results:**

Field-grown protocorms broadly aligned with the in vitro symbiotic model, while also showing how environmental signals refine a shared core transcriptional programme. Both symbiotic conditions showed transcriptomic patterns compatible with fungal colonisation but differed in the relative contribution of regulatory, transport, and stress-associated functions. Field protocorms showed stronger activation of membrane transport and wall-associated mechanosensing pathways, while sustaining defence-related regulation at a restrained, metabolically integrated level. Under in vitro conditions, symbiotic protocorms showed enhanced activation of stress-responsive and detoxification pathways compared with field-grown ones. The expression of light-associated plastid differentiation genes was attenuated despite brief culture-related illumination. In contrast, asymbiotic protocorms showed stronger activation of light- and oxidative-stress-related responses.

**Conclusions:**

Together, these patterns indicate that field-grown protocorms show a more integrated and environmentally modulated transcriptional organisation, whereas in vitro symbiosis is associated with stress buffering and compensatory metabolic responses. By integrating field and laboratory datasets, this work provides a more complete portrait of early orchid symbiosis and protocorm physiology, clarifying both the interpretive power and limitations of current in vitro models.

**Supplementary Information:**

The online version contains supplementary material available at https://doi.org/10.1186/s12870-026-09554-y.

## Background

Orchid-fungus mycorrhizal interactions exhibit remarkable diversity in terms of fungal partners, nutritional strategies, ecology and biogeography [1–3]. All orchids pass through a protocorm stage early phase of their life cycle. The protocorm is an undifferentiated structure that hosts fungal hyphae forming densely coiled pelotons in some of its cells [4–6]. The transfer of water and nutrients, including carbon, from the fungus to the plant is essential for the development of the reserve-poor embryo [7, 8]. This nutrition is called mycoheterotrophy [9, 10]. In adulthood, most orchids shift to autotrophy, while a few species remain achlorophyllous and thus mycoheterotrophic [11, 12]. Despite representing a compelling research subject, particularly in the context of mycoheterotrophy, orchid mycorrhiza remains comparatively understudied compared with arbuscular mycorrhiza or nitrogen-fixing legume symbiosis [13, 14].

Recent developments in molecular omics technologies have significantly advanced the field [15–17], with RNA sequencing (RNA-seq) being particularly valuable for providing genome-wide, unbiased gene expression profiling [18]. These approaches have shown that although orchid mycoheterotrophic symbiosis has long been considered a form of plant parasitism on fungi (‘epiparasitism’) [19], it shares various mechanisms involved in the signalling, establishment, and maintenance of typically mutualistic arbuscular mycorrhizal and nitrogen-fixing rhizobial interactions [20–23]. Nonetheless, some features unique to orchid mycorrhizas have been revealed, including the distinct regulation of seed germination through gibberellin-dependent control of orchid-mycorrhizal symbiosis [24, 25]. Alongside these findings, new concepts regarding mechanisms of nutrient exchange between mycoheterotrophic plants and fungi are now emerging [26].

In vitro cultures with fungi or without fungi (asymbiotic controls) are the most frequently used model for studying mycoheterotrophic symbiotic germination in orchids [e.g. 16, 27, 28]. Although these controlled experiments allow detection of responses to specific factors, they provide limited ecological realism and may introduce experimental artefacts [29, 30]. These constraints are highlighted by differences in morphology, anatomy, and in germination potential between field- and in vitro-grown protocorms [31, 32]. Furthermore, different media requirements for symbiotic and asymbiotic growth complicate the comparison and interpretation of data [20, 33]. In vitro cultivation conditions do not fully capture the complexity of biotic interactions in natural habitats. For instance, Read et al. [34] demonstrated, in a complex microcosm experiment, that adult orchids transfer carbon to protocorms *via* shared mycorrhizal hyphae. Thus, in natural settings, mycorrhizal networks associated with mature photosynthetic plants may serve as an important carbon source for developing protocorms, a source that is absent under standard in vitro culture conditions. These findings are consistent with the broader and still poorly understood ecology of orchid-associated ‘rhizoctonia’ fungi, which are routinely used in in vitro experiments. Beyond their well-documented saprophytic abilities [1], these fungi may also occur as endophytes in the roots of non-orchid plants [2, 35].

Protocorms developing under natural conditions may form a more functionally dynamic association with mycorrhizal fungi, shaped by local biotic and abiotic factors [33, 36]. Although environmental conditions in natural habitats can be suboptimal, interactions with a broader microbial community are known to improve resource availability and to reduce stress in plants [37–39]. Field-grown protocorms may ultimately experience different (more or less efficient) nutrient provisioning, including nitrogen and phosphorus, and be exposed to stress factors that differ from those encountered in vitro. However, the physiological response of protocorms grown in nature remains untested; investigating this response would provide deeper insight into the ecological and evolutionary dynamics of orchid mycorrhizas.

Here, we present the transcriptomic landscape of protocorms of the temperate terrestrial orchid *Dactylorhiza majalis* (Rchb.) P.F.Hunt & Summerh, grown in the field under natural symbiotic conditions (FS), as well as in vitro under either controlled symbiotic conditions (IVS) with compatible *Tulasnella* fungi [40] or asymbiotic conditions (IVA), a well-established model in in vitro orchid studies [e.g., 41, 42]. This design allowed us to disentangle the effects of the growth environment (field vs. in vitro) from those of symbiotic status, while recognising that asymbiotic development occurs exclusively under in vitro conditions.

We aimed to explore the molecular mechanisms of orchid symbiotic germination, while assessing the limitations of the in vitro model. We focused particularly on differences in transcriptomic profiles between orchid protocorms grown symbiotically in the field and in vitro with respect to the expression of genes (1) involved in nutrient and water transport, interpreted as indicators of resource-capture efficiency, (2) related to metabolism and biosynthesis, interpreted as indicators of resource utilisation, (3) related to partner recognition and symbiosis maintenance, and (4) involved in response to stress, whose expression was expected to differ across conditions.

## Methods

### Collection, provenance and allocation of *D. majalis* seeds

The western marsh orchid, *D. majalis*, is a tuberous perennial orchid belonging to the subtribe Orchidinae within the subfamily Orchidoideae [43]. It is found in wet meadows, marshes, and fens across Europe. The species is known to associate with rhizoctonias across its entire distribution range [40, 44]. Materials for the study were collected in May 2017 in the two natural populations of *Dactylorhiza majalis* in southern Poland: Psary (50.368, 19.081) and Trzebyczka (50.396, 19.361). Species identification was performed by Julita Minasiewicz. Both populations were initially included to obtain local seed material and root-derived fungal isolates and to establish a symmetric design in which seeds were returned to their population of origin for field baiting. However, fruit set, germination success and recovery of protocorms suitable for RNA-seq differed between populations and growth conditions. Consequently, the final dataset was not population-balanced: in vitro protocorms were obtained from seeds collected in Psary, where fruit set was higher, but no field protocorms were recovered from this population. In Trzebyczka the lower amount of available seed material was preferentially allocated to the field-baiting experiment, from which the FS protocorms were eventually recovered. Details of sample provenance are provided in Supplementary Table S1.

Seed capsules, 30–50 in each population, were collected in 2017, and air-dried at room temperature. They were opened on a glass Petri dish, and capsule residues were then removed manually with forceps. Seeds were pooled only within populations. Each population-specific seed pool was then divided into two groups: one for the in vitro and one for the field experiments. Seeds assigned for in vitro experiments were stored in dark, dry conditions at + 4 °C for several months. This short-term storage under cold, dry conditions was used to maintain seed material until fungal isolation and preparation for the laboratory germination experiments had been completed. Seeds assigned to field experiments were placed in mesh bags and returned to their source population in 2017, the year in which the capsules were collected. Voucher material of the species was deposited in the Herbarium Universitatis Gedanensis (UGDA:0053279–0053282).

### Field seed sowing and protocorm collection

The orchid seeds-sowing technique [45] was used to obtain field-grown symbiotic protocorms. Nylon-mesh (50 μm, Surtex, Poland) bags, 20 cm in length and 5 cm in width, were divided into six cells, each 3 cm in length. Approximately 200 seeds were put into each cell. The bags were inserted vertically into the soil no further than 30 cm from mature plants of *D. majalis* in the corresponding seed source populations, Psary and Trzebyczka, in the year the seeds were collected. The seed packets were checked annually for germination progress. Three field biological replicates were collected in July 2019 and one in July 2021 (Supplementary Table S1). A collection pipeline was established in the field, at a sheltered collection station. Seed bags were gently excavated and washed before opening to remove soil debris. They were opened on a Petri dish and examined under a stereoscope and quickly screened for protocorms at developmental stages 3 and 4 according to Tešitelová et al. [46]. Selected protocorms were gently transferred with forceps into a second Petri dish filled with clean water and placed under another stereoscope for thorough final cleaning. Then, they were transferred into the Eppendorf tubes and flash-frozen in liquid nitrogen (Supplementary Fig. S1). The procedure was optimised to obtain maximum protocorm cleanliness with minimal time and disturbance and did not take longer than 10 min for each batch. Eppendorf tubes were transferred from liquid nitrogen into an ultracold freezer and kept at -80 °C until further processing. Voucher specimens of protocorms and seeds were not deposited because they could not be reliably preserved as herbarium specimens.

### Roots collection and fungal strain isolation and testing

For fungal isolation, one root was collected from each of four adult *D. majalis* plants in each population. Roots were thoroughly washed, wrapped in moist tissue, placed in a zip-lock bag, and kept cool during transport to the laboratory. Upon arrival, the roots were immediately processed to isolate symbiotic fungal strains following the procedure described by Warcup and Talbot [47]. The obtained fungal isolates were first selected based on colony and hyphal morphology consistent with that of orchid-associated ‘rhizoctonia’ fungi [47]. The fungal isolates finally used for the IVS treatment originated from adult *D. majalis* roots collected in the Psary (DM_PSAR_14B) and Trzebyczka (DM_TRZE_19) populations (Supplementary Table S1) and were selected based on their ability to support *D. majalis* seed germination and protocorm development. Precultures of these isolates were maintained on potato-dextrose agar (PDA).

MiCoP analysis [48] was subsequently performed on the RNA-seq data to identify the fungal taxa detected in the sequenced IVS and FS samples and to compare the fungal component between the two symbiotic conditions. This method was used to ensure comparability between the field-grown and *in vitro-*grown symbiotic protocorms, as the sample size of the former did not allow separate DNA-based molecular barcoding analyses. MiCoP analysis showed that IVS and FS shared the same fungus (*Tulasnella irregularis* Warcup & P. H. B. Talbot). Three additional *Tulasnella* species were identified in FS protocorms (*T. cumulopuntioides* S. Fujimori, J.P. Abe, I. Okane & Y. Yamaoka; *T. epidendrea* Nkomo, Hammerb., B.D. Wingf. & T. Bose and *T. brigadeiroensis* E.F.S. Freitas, Meir. Silva & M.C.M. Kasuya) (Supplementary Table S2).

### In vitro symbiotic and aymbiotic seed germination

The seeds were disinfected in syringes according to Ponert et al. [49]. Subsequently, they were incubated for five minutes in 70% ethanol, washed with deionised water, incubated for five minutes in a sterilising solution prepared from chlorinated lime (10 g in 50 mL of deionised water), washed with sterile deionised water, and then dispensed as a suspension onto the surface of solidified medium in 9-cm plastic Petri dishes.

For symbiotic experiments, we used a simple OMA medium as described previously [41]. This medium contained 1% agar (w/v, Sigma-Aldrich) and 0.3% oatmeal powder (Himedia). For asymbiotic germination, the sugar-amended complex ¼-2 medium was used [49]. In this case, the pH was adjusted to 5.8 using KOH and HCl. Both media types were autoclaved at 144 kPa and 121 °C (Tuttnauer 2540 EK-N) for 20 min and poured into 9-cm plastic Petri dishes. After seed sowing, an agar plug (approximately 0.5 cm²) with the mycelium of the selected fungal isolate was placed at the centre of each Petri dish containing OMA medium, except for the control plates, which were treated with mycelium-free plugs. Subsequently, the dishes were sealed with air-permeable foil (Parafilm M) and kept in the dark at 19 °C. Based on our prior experience with *D. majalis* [41], cultures (Supplementary Fig. S2) were monitored every two weeks from the second month after sowing using a Zeiss Stemi 305 stereomicroscope. Each monitoring event exposed the seeds/protocorms to ca. 30–45 min of illumination.

Protocorms at developmental stages 3–4, as defined by Tešitelová et al. [47], were selected and transferred into Eppendorf Safe-Lock tubes, which were immediately plunged into liquid nitrogen. The samples were subsequently stored at − 80 °C until further processing. We chose a developmental stage-based method rather than an age-based one, since the exact age of field-grown protocorms was unknown, and this method allows protocorms at similar developmental stages to be compared in both experiments.

### RNA extraction and purification

All protocorms of *D. majalis* collected from the three experimental conditions (field, in vitro asymbiotic, and in vitro symbiotic) were subjected to the same RNA extraction procedure. Each single-protocorm sample was homogenized in liquid nitrogen using a TissueLyser II in 2 mL Eppendorf tubes containing metal beads. RNAs were extracted using NucleoZOL according to the manufacturer’s protocol (Macherey-Nagel, Düren, Germany) with polyvinylpolypyrrolidone added during grinding to remove phenolics, followed by further purification using an RNA Cleanup and Concentration Kit (Macherey-Nagel, Düren, Germany).

RNA integrity was checked on a Plant RNA Nano Chip using the Agilent 2100 Bioanalyzer (Agilent Technologies, Waldbronn, Germany). RNA concentrations were measured using a RiboGreen assay (Thermo Fisher Scientific, Waltham, MA, United States). If RNA quantity was insufficient, samples from the same experimental condition were pooled to make one biological replicate. Four biological replicates were generated for each of the three conditions. Each replicate consisted of 1–2 protocorms (see Supplementary Table S1).

### Illumina sequencing and quality trimming

The libraries were constructed using the TruSeq Stranded mRNA Kit (Illumina, San Diego, CA, United States). They were then sequenced on a NextSeq 500 (Illumina, San Diego, CA, United States) platform in single-end mode with a read length of 75 bases. After quality trimming with Trimmomatic (v0.36, parameters: SE and ILLUMINACLIP: TruSeq3-SE.fa:2:30:10:2:true LEADING:21 TRAILING:21 MINLEN:30 [50]), residual ribosomal RNA reads were filtered out with SortMeRNA v2.1 (against the databases silva-bac-16s-id90, silva-bac-23s-id98, silva-euk-18s-id95, and silva-euk-28s-id98 with the parameters – e 1e-07 – paired_in). After filtering, between 12.8 M and 30.4 M (average and median: 22.8 M) per sample were generated.

Sequencing read libraries were mapped against the *D. majalis* transcriptome assembly available at the INRAE Dataverse under the DOI: https://doi.org/10.57745/NSPILL.This This assembly and the filtering out of fungal symbiont transcripts were performed as described in Jakalski et al. [17] (the full dataset will be published elsewhere). The read mapping process was performed using BBMap from the BBTools package, v.38.22 [51]. The software was run with the additional “rpkm” parameter, which yields per-contig raw counts directly alongside the standard SAM/BAM output files, so that a raw count matrix was generated. Between 9.3 M and 23.1 M reads per sample (average and median: 15.1 M) were mapped and counted corresponding to a mapping rate of about 75% for asymbiotic samples and 65% for symbiotic ones, as expected.

### Statistical analyses

#### Expression data

Statistical analyses were conducted in R v4.3.1 [52] using the R script-based tool DiCoExpress [53, 54] based on the Bioconductor package edgeR v 4.0.16 [55, 56].

#### Gene filtering and normalization

Genes with low counts were filtered using the “filterByExpr” function, where the group argument specified the biological conditions, the min.count value was set to 5, and the min.total.count, large.n, and min.prop arguments are set to their default values. Libraries were normalized with the Trimmed Mean of M-values (TMM) method using the function “calcNormFactors” with the default parameter values.

#### Differential expression analysis

The differential analysis was based on a negative binomial generalized linear model in which the logarithm of the average gene expression was a function of the condition effect: field symbiotic (FS), in vitro symbiotic (IVS) and in vitro asymbiotic (IVA). Based on the experimental design, DiCoExpress [53] automatically generated three contrasts corresponding to the three pairwise comparisons of the three conditions. For each contrast, a likelihood ratio test was applied, and raw p-values were adjusted with the Benjamini-Hochberg procedure [57] to control the false discovery rate. The distribution of the resulting p-values followed the quality criterion described previously [58]. A gene was declared differentially expressed if its adjusted p-value was lower than 0.05. Functional classification of differentially expressed genes (DEGs) was based on functional enrichment analyses performed within DiCoExpress using annotations identified with TRAPID [59], and Mercator4 v7.0 [60].

#### Selection of representative genes

For gene-level interpretation, we focused on candidates from enriched functional categories that corresponded to the main research aims (transport, metabolism, symbiosis maintenance, and stress responses). This strategy ensured that the gene-level discussion was biologically grounded and consistent with the research questions outlined in the introduction.

#### Relative classification of gene expression patterns

To compare symbiotic responses under field and in vitro conditions, we first identified the DEGs of the FS/IVS contrast and then characterized their field (FS/IVA) and in vitro (IVS/IVA) responses using IVA as a common reference. For each FS/IVS DEG, we first considered the direction of regulation (up- or downregulation) in the FS/IVA and IVS/IVA comparisons. Genes regulated in the same direction in both contrasts were then classified according to the relative magnitude of their response, based on log₂ fold-change values. A field response was classified as stronger when |log₂FC| was greater for FS/IVA than for IVS/IVA, and as weaker when |log₂FC| was smaller for FS/IVA than for IVS/IVA. For example, RSU genes were upregulated in both FS/IVA and IVS/IVA but showed stronger upregulation in FS/IVA; RWU genes were also upregulated in both contrasts but showed weaker upregulation in FS/IVA. The same logic was applied to downregulated genes, producing RSD and RWD classes. Genes regulated in opposite directions between FS/IVA and IVS/IVA were assigned to inversion classes: IUD for genes upregulated in FS/IVA and downregulated in IVS/IVA, and IDU for the reverse pattern. These classes of FS/IVS DEGs were used as an integrative framework to describe expression patterns across FS, IVS, and IVA, rather than as additional pairwise contrasts. Thus, RSU and RWU denote relative stronger and weaker upregulation in the field response, respectively; RSD and RWD denote relative stronger and weaker downregulation in the field response, respectively; whereas IUD and IDU denote the two inverted regulation classes.

## Results

### Impact of growth mode on gene expression of in vitro protocorms

**Fig. 1:**
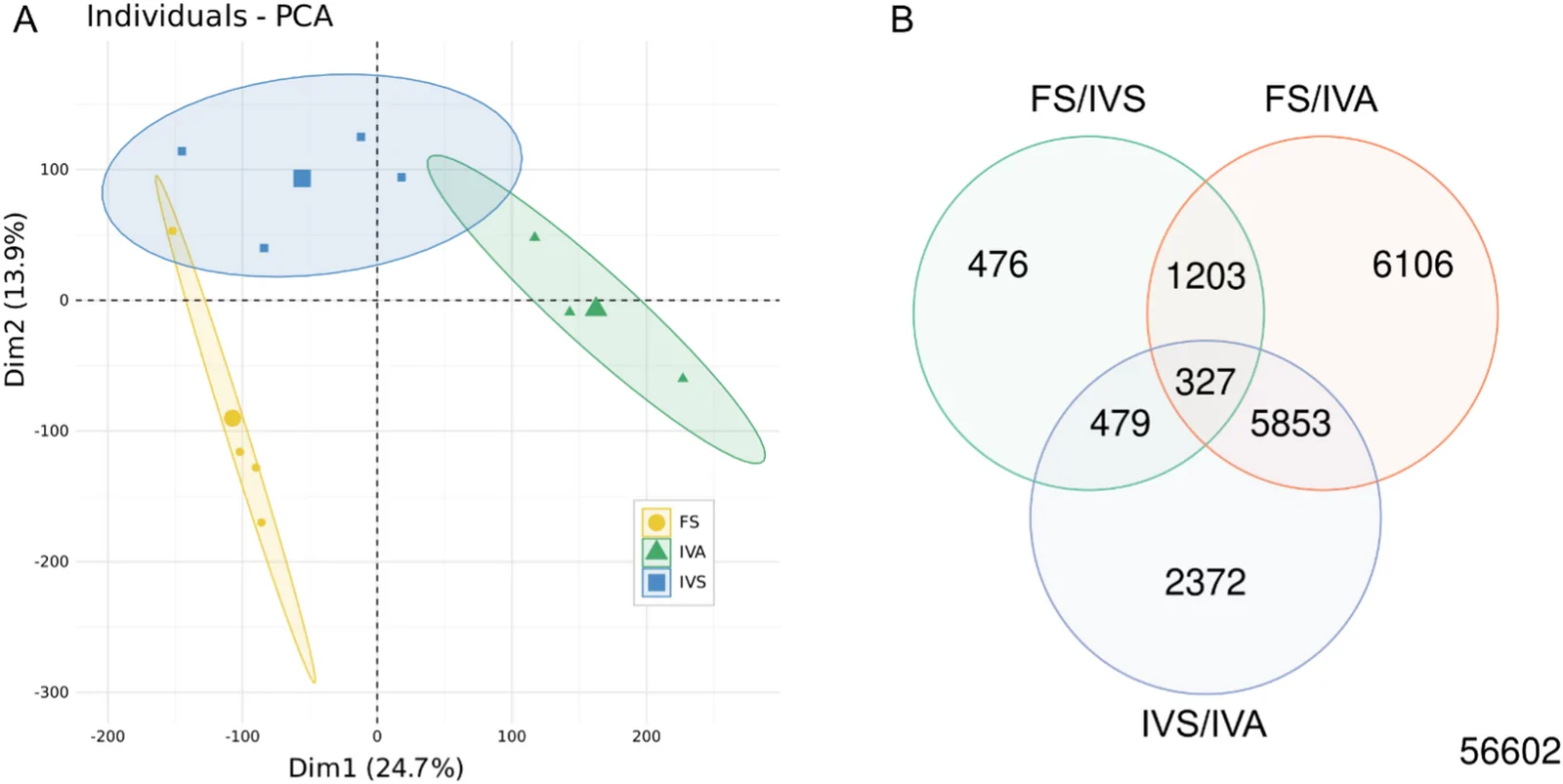
Statistical analysis of the annotated and differentially expressed unigenes (DEGs) in *D. majalis* protocorms comparisons. **A** Principal Component Analysis based on RNA-seq data from D. majalis protocorms: FS - field symbiotic (yellow); IVS - in vitro symbiotic (blue); and IVA - in vitro asymbiotic (green). The ellipses represent the 95% confidence interval and the larger symbol within each ellipse is the barycentre (**B**) Venn diagram of all DEGs between respective comparisons: field symbiotic (FS), in vitro symbiotic (IVS) and in vitro asymbiotic (IVA) protocorms. The number outside the Venn diagram is the number of genes analysed but not detected as DEG in any of the three comparisons

We first used PCA (Fig. 1A) to provide an overview of transcriptomic separation among the three conditions, and a Venn diagram (Fig. 1B) to summarize the shared and comparison-specific DEG sets across the three pairwise comparisons. The PCA diagram (Fig. 1A) clearly separated symbiotic and asymbiotic protocorms based on gene expression profiles along the first axis (24.7% of the total variance). We identified 9,031 DEGs between IVS and IVA protocorms among which 5,423 were upregulated by symbiosis (i.e. higher in IVS) while 3,608 only were downregulated. To characterise these gene lists, we identified 429 overrepresented GO, Mapman, and KEGG functions among genes upregulated in IVS protocorms, and 1,587 functions among genes downregulated in IVS protocorms (FDR < 0.05; Supplementary Table S3).

**Fig. 2:**
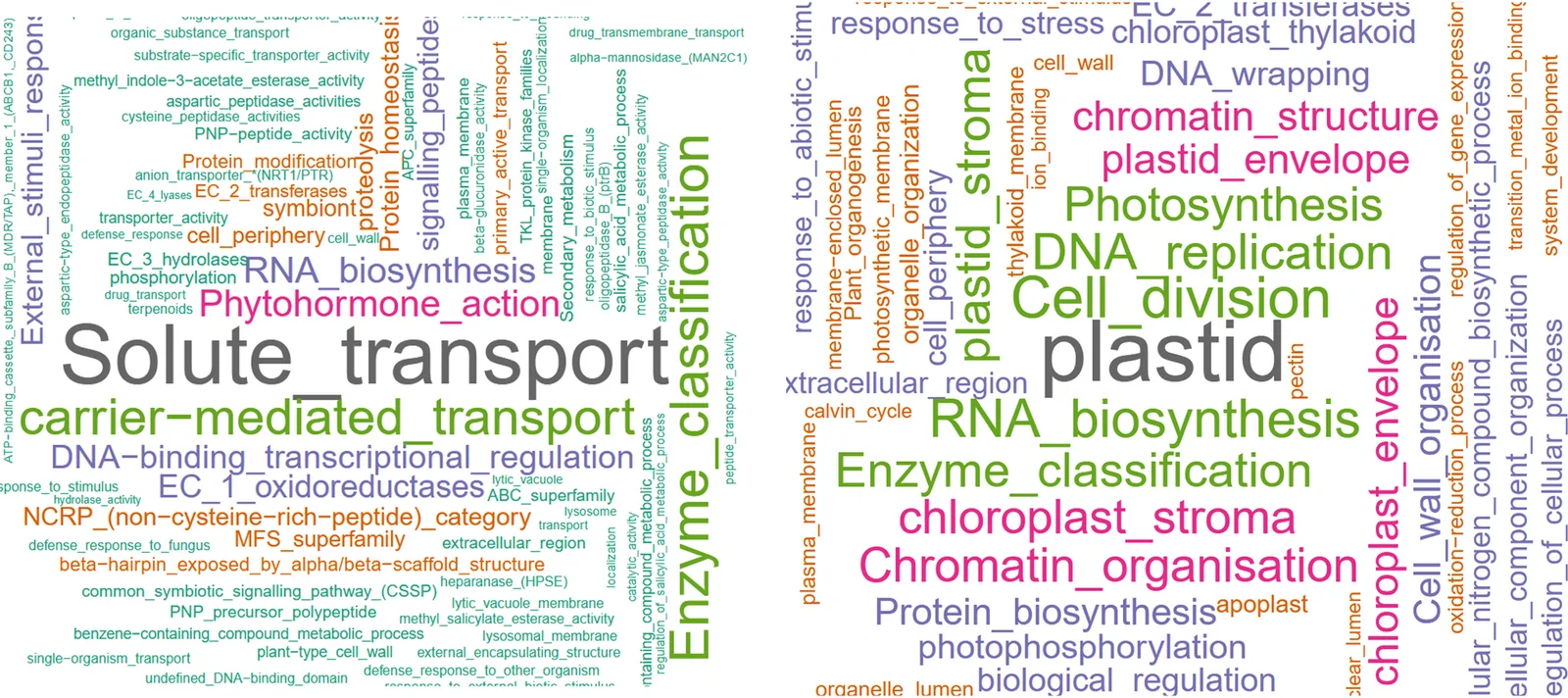
Word clouds representing the relative frequency of functions significantly enriched in the comparison of in vitro protocorms of *D. majalis*: symbiotic (IVS) versus asymbiotic (IVA). The left graph shows functions enriched in genes upregulated in IVS; the right graph shows functions enriched in genes upregulated in IVA. Functions that appeared more frequently in the analysis are displayed in proportionally larger font size. The word clouds provide a compact representation of dominant enriched functions from Supplementary Table S3

Enrichment analysis revealed general downregulation of functions connected with plastids in IVS (Fig. 2). Upregulated functions included the core components of the common symbiotic signalling pathway, including DELLA, GRAS-type regulators, and CYCLOPS, but also multiple carrier-mediated transport systems, such as SWEET, amino-acid and peptide transporters, together with the.

membrane-trafficking component SNAP25. Protein-processing-related categories showed broad upregulation, encompassing peptidases, ubiquitination-related factors, protein-modifying enzymes, and protein quality control. Carbohydrate-related functions associated with symbiosis showed increased representation of trehalose-related metabolism and β-glucosidase activity, whereas genes involved in starch metabolism and lipid storage were downregulated. Hormone-related enrichment included induction of rapid auxin-responsive genes (SAURs), increased gibberellin turnover via *GA2ox*, and Ca²⁺-sensor proteins (CML). Secondary metabolism upregulation in IVS included phenylpropanoid and terpene pathways, defence-related enzymes, and UDP-glucosyltransferases, accompanied by oxidative monooxygenases linked to specialised-metabolite biosynthesis. Functions associated with glucosinolate and phytoalexin metabolism were also upregulated. Relative to asymbiotic protocorms, which were enriched in ascorbate- and thiol-based antioxidant functions, symbiotic protocorms showed enrichment of redox-related functions associated with detoxification,

including glutathione biosynthesis and ABC transporter–mediated processes. Whereas *HY5* (ELONGATED HYPOCOTYL 5), a central regulator of photomorphogenesis, was downregulated in symbiosis, upregulated transcription-factor families included NAC, WRKY, ZAT, and IDD families, in line with broader enrichment of global transcriptional-regulation categories. Cell-wall-related functions were predominantly downregulated, while IVS protocorms displayed concurrent enrichment of mechanosensing and peptide-signalling components, including WAK/WAK-like regulators and other receptor-like elements.

### The response to symbiosis was shared by the field and in vitro protocorms

The PCA analysis (Fig. 1A) showed that symbiotic samples largely overlapped along the first dimension and were separated from asymbiotic samples. This pattern suggests that field-grown (FS) and in vitro symbiotic protocorms (IVS) had largely similar but not identical transcriptomic profiles.

When focusing on genes detected as differentially expressed in both symbiotic contrasts (FS/IVA and IVS/IVA), we identified 6,180 genes representing a shared core of the symbiotic response (Fig. 1B). Among these, 99.6% (6,155 DEGs) varied in the same direction in both the field and in vitro responses, being either consistently up- or downregulated. This strong concordance was not limited to genes differentially expressed in both contrasts. When all genes detected as differentially expressed in at least one of the two contrasts were considered (16,340 genes in total), 94.2% (15,399 genes) likewise showed the same direction of regulation across field and in vitro responses. Together, these results show that transcriptional responses associated with fungal symbiosis were largely concordant between in vitro and field-grown protocorms.

However, the two symbiotic protocorm groups (i.e. FS and IVS) showed visible separation in the PCA (Fig. 1A) according to growth environment, and 2,485 genes were differentially expressed between these treatments. This number was much lower than that for the comparison of FS with IVA (13,489 DEGs) or IVS with IVA (9,031 DEGs) (Fig. 1B), yet it supports clear differences between field-grown and in vitro-grown protocorms. To further characterise these differences, we used the relative classification framework described in the Methods and found that they fell into two broad expression groups of comparable size (1,195 and 1,290 genes).

**Fig. 3:**
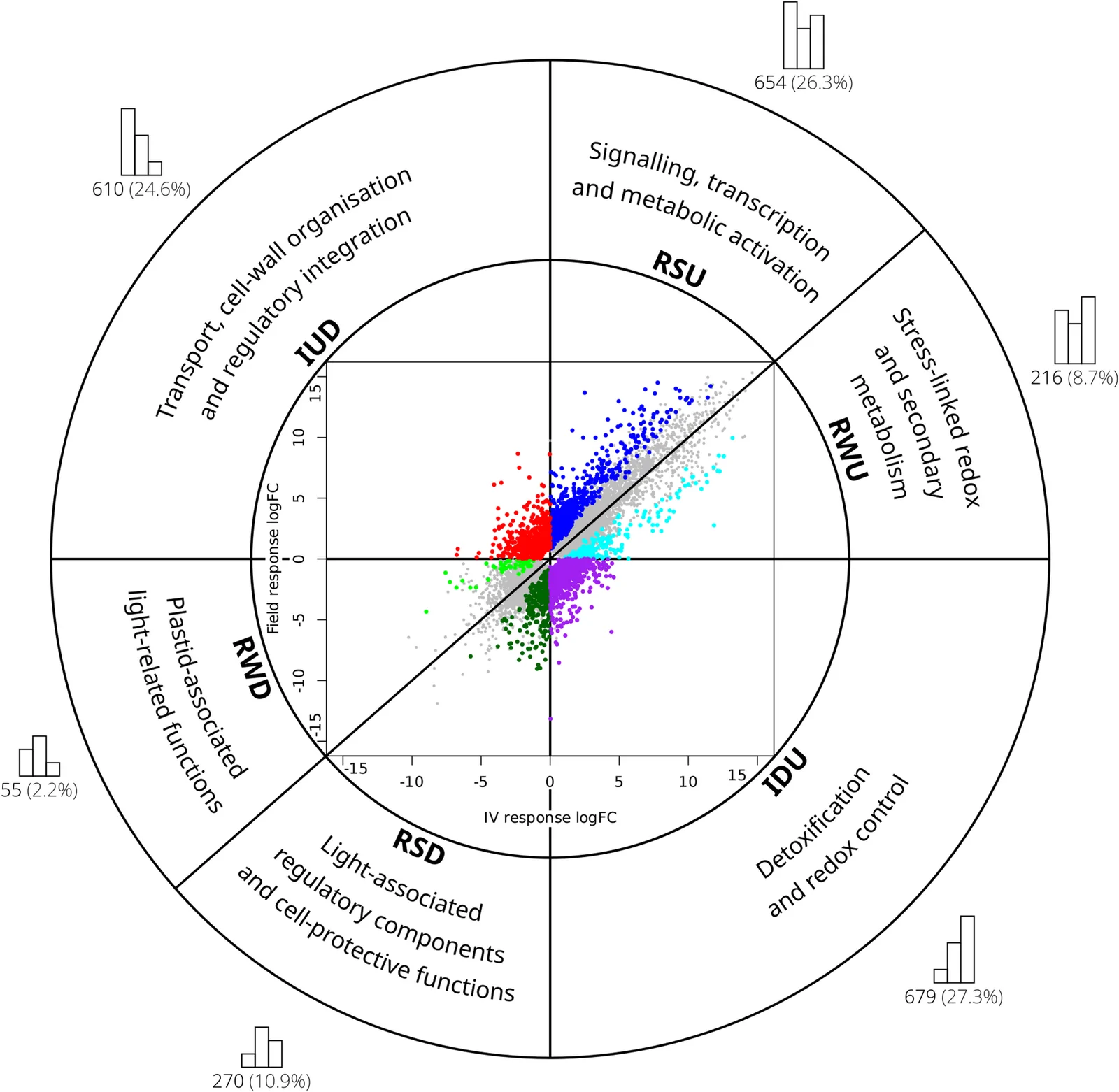
Environmental modulation of the symbiotic response in *D. majalis* field symbiotic (FS), in vitro symbiotic (IVS) and in vitro asymbiotic (IVA) protocorms. The central scatterplot shows RNA-seq log₂ fold changes for all analysed genes, comparing field symbiotic (FS/IVA; vertical axis) and in vitro symbiotic (IVS/IVA; horizontal axis) responses. Each point therefore represents the relative magnitude and direction of the symbiotic response under field and in vitro conditions for the same gene.Coloured dots denote the 2,485 differentially expressed genes (DEGs) between FS and IVS, whereas grey dots represent the remaining analysed genes. Blue and cyan dots denote upregulated genes; green and dark green downregulated genes; violet/red - inversely regulated genes between the two symbiotic responses. Within the same-direction classes, stronger regulation in FS relative to IVS is shown in blue and dark green, whereas weaker regulation in FS relative to IVS is shown in cyan and green.The outer ring is divided into six DEG classes (RSU, RWU, RSD, RWD, IUD, IDU) based on their relative behaviour between FS/IVA and IVS/IVA contrasts. Functional themes assigned to each class are based on enrichment analyses (FDR < 0.05).Side bars show illustrative expression patterns for each class, presented in a consistent FS–IVA–IVS order. Numbers below the bars indicate the number of genes in each class and their proportion within the set of 2,485 DEGs between FS and IVS. One of the 2,485 DEGs (TRINITY_MR_DN85670_c2_g2_i1) is only expressed in FS and so it could not be assigned to any class. Detailed enriched functions for each class are provided in Table S4.RSU - relative stronger upregulation; RWU - relative weaker upregulation; RSD - relative stronger downregulation; RWD - relative weaker downregulation; IUD - inverted regulation (upregulated in FS/IVA and downregulated in IVS/IVA); IDU - inverted regulation (downregulated in FS/IVA and upregulated in IVS/IVA)

The first group comprised genes showing the same direction of response, being either up- or downregulated in both the field and in vitro responses, but differing in the magnitude of the response (Fig. 3). Within this group, 924 genes (77%) exhibited relatively stronger responses in the field response (RSU/RSD), whereas 271 genes (23%) showed relatively weaker responses in the field (RWU/RWD). Importantly, this predominance of stronger field responses extended to the 16,340 genes differentially expressed in either FS/IVA or IVS/IVA: 10,628 of them (65%) showed the same direction of response but a greater magnitude in FS/IVA than in IVS/IVA. Hence, fungal symbiosis was associated with broadly concordant transcriptional responses in vitro and in the field, but the response more often showed greater magnitude in the field.

The second group consisted of genes showing inverted responses between the field and in vitro conditions (IUD/IDU), representing a distinct set of growth-context-associated differences within the symbiotic responses. However, it should be noted that only 25 genes with inverted responses were significantly differentially expressed in all three comparisons, confirming the strong similarity of transcriptional responses to symbiosis under in vitro and field conditions. To further explore the functional basis of these patterns, we next analysed the enriched GO, KEGG and MapMan categories, as summarised in Supplementary Tables S4 and S5 and presented below.

### Natural environment enhanced transport, structural integrity, and stress-developmental regulation

The transcriptional profile of the field response showed a concurrent activation of transport, signalling, and regulatory pathways. The IUD class showed strong enrichment of genes related to solute transport, cell wall organisation, and transcriptional regulation. The stronger-upregulated gene class (RSU) complemented these trends with additional transcription-factor genes, signalling modules, and protein-turnover components (Fig. 3; Table 1, Supplementary Tables S4, S5).

**Table 1:** Functional modules with representative enriched functions and top driver genes across expression classes. FS, field-grown symbiotic protocorms; IVS and IVA, in vitro symbiotic and asymbiotic protocorms, respectively. Expression classes were defined based on the relative magnitude and direction of transcriptional regulation inferred from FS/IVA and IVS/IVA contrasts: RSU, relative stronger upregulation in FS; RWU, relative weaker upregulation in FS; RSD, relative stronger downregulation in FS; RWD, relative weaker downregulation in FS; IUD, inverted regulation (upregulated in FS/IVA and downregulated in IVS/IVA); I IDU, inverted regulation (downregulated in FS/IVA and upregulated in IVS/IVA). Functional annotations and enriched terms were assigned using MapMan, KEGG, and Gene Ontology (GO). See Supplementary Table S5 for details

Expression classes	Module	Representative enriched function(s)	Top drivers	Possible role
IUD	Solute & water transport	GO:0051119; MapMan 24.2; K09872	SWEETs, *UMAMIT*,* AAP/AAAP*,* PIP*,* PHO/PHT*	Enhanced amino-acid, sugar, ion and water flux characteristic of FS
IUD	Cell wall remodelling & cell wall integrity signalling	MapMan 21.1; GO:0009505	*COBRA*,* GATL*,* XTH*,* CrRLK1L*,* ASAH2*	Cell-wall remodeling and signalling characteristic of FS
IUD	Hormone/redox-linked transcriptional regulation	MapMan 15.5	TIFY (JA repressors), ERF-VIII/X, NAC, MYB22/23/27, bHLH III/VIII	Hormone/redox transcriptional set characteristic of FS
RSU	Transport (UmamiT-specific)	GO:0032973	UmamiT	Stronger activation of amino-acid transport in FS
RSU	Apoplastic & peptide signalling	MapMan 11,1; GO:0007167	PSK/PNP peptides, LRR-III RLKs, CMLs, SAURs	Strengthened extracellular perception and Ca²⁺-linked signalling
RSU	Transcriptional coordination	MapMan 15	WRKY, NAC, ZAT, bHLH, HD-ZIP, DOF	Integration of growth, stress and metabolic regulation
RSU	Protein turnover & quality control	K16292, K00737,	Ubiquitination enzymes, peptidases	Enhanced proteostasis associated with field metabolic demand
IDU	Detoxification & oxidative metabolism	K13496; MapMan 10.2 & 24.1.3	CYP450, GSTs, UGTs, ABC transporters	IVS-specific induction of detoxification/oxidation block
IDU	Plastidic redox & nitrogen metabolism	MapMan 25.1, K00284, K00099	*NIR1*,* Fd-GOGAT*,* DXR*	Activation of plastidic redox/isoprenoid metabolism in IVS
IDU	Phenolic & oxylipin turnover	MapMan 11.7; GO:0009505, K13496	*SCPL19/SNG2*,* UGTs*,* OPR4*	Oxylipin/phenolic conjugation and modification in IVS
IDU	light induced TFs	MapMan 15	PIF1, FRS/FRF	red/far-red light signalling and suppression of light signalling in IVS
IDU	Stress-responsive transcriptional modules	MapMan 15	WRKY, NAC, HSF	Broad stress-TF activation defining IVS symbiosis
IDU/RWU	Membrane/lipid-stress signature of IVS	MapMan 24, K10258	*ALA (P4-ATPase)*,* ECR*	Membrane/lipid-stress signature of IVS
RWU	Detoxification & conjugation	MapMan 50	GSTs, UGTs, ABC transporters, CYP450	Higher detoxification activity in IVS
RWU	Hormone-linked stress signalling	MapMan 50	GH3.8, ACC oxidase	Higher auxin conjugation, ethylene biosynthesis in IVS
RWD	Plastid-associated components	K13783, GO:0010287, GO:0016020	PSBO/OEC33, LHCa1/3, LCY-e	Stronger suppression plastid-associated genes in IVS
RSD	Light-associated regulatory components	K10775; K10775, MapMan 26.1, GO:0009987	HY5, UV-responsive genes, *PAL*,* HCF244–OHP*	Strong repression of light regulatory genes under natural soil darkness
RSD	genome-protection attenuation	GO:0009411, GO:0009987	*PHR1*,* POT1a*,* GEN1*	Stronger suppression of genome protective pathways in FS

In the IUD class, solute transport functions were dominated by SWEET carriers and several amino-acid transporter families (UmamiT, AAP, AAAP), together with PIP aquaporins and ion-transport systems, including phosphate-related PHO and PHT transporters.

Although cell wall-related functions were generally downregulated in symbiosis, the field response retained a distinct IUD class of canonical enzymes involved in sugar and cell-wall remodelling combined with multiple cell wall integrity (CWI)-associated signalling and regulatory components. These included structural wall-related regulators such as *COBRA*, neutral ceramidase/*ASAH2* and a CrRLK1L-type receptor. Although the last gene was formally assigned to RSU, it is discussed here because its classification was mainly driven by upregulated in FS, whereas its upregulation in the IVS–IVA difference was marginal. In addition, the strongest RSU signals in the field response were associated with apoplastic peptide signalling, including PNP- and PSK-like ligands together with their LRR-III-type receptor kinases, Ca²⁺-responsive CML sensors, and auxin/mechanosensitive SAUR family genes (Table 1, Supplementary Table S5).

Transcriptional regulation was prominent among field-response-specific IUD genes. Whereas the redox- and stress-centred TF repertoire (WRKY, NAC, ZAT) was upregulated in both symbiotic responses, the field response showed enrichment in hormone-linked TIFY-family repressors and ERF class VIII/IX factors, together with MYB-family metabolic regulators (e.g., *MYB22/23* and *MYB27/48/59*). The dataset also contained developmental TF families, including bHLH, HD-ZIP, and DOF groups associated with hormonal and light-associated signalling, as well as KNOX, BEL, GRF, and E2F classes linked to meristem and cell-cycle regulation (Fig. 3; Table 1, Supplementary Table S5). Overall, the field response was enriched in TF families related to growth regulation, metabolic adjustment, and signal integration. Together, the field-response-specific IUD and RSU profiles highlighted transport, peptide/Ca²⁺-linked signalling, cell-wall-associated regulation, and transcriptional control.

### Integrated stress-detoxification response defined the in vitro symbiotic transcriptome

The specificity of the in vitro symbiotic response was reflected in the concurrent activation of multiple stress-associated genes, which were grouped into five sets within the IDU and RWU classes (Fig. 3; Table 1, Supplementary Tables S4, S5). First, a broad detoxification core was induced, including cytochrome P450 monooxygenases, several glutathione S-transferases, UDP-glycosyltransferases (UGTs), and ABC transporters. These enzymes formed an oxidation–conjugation–export block consistently enriched in both the IDU and RWU classes. In addition, four IDU-restricted gene sets were identified. The first set comprised plastid-linked redox-related genes including nitrite reductase (*NIR1*), ferredoxin-dependent glutamate synthase (*Fd-GOGAT*) and 1-deoxy-D-xylulose-5-phosphate reductoisomerase (*DXR*), consistent with plastid-associated nitrogen and isoprenoid redox metabolism. The second involved phenolic and oxylipin turnover, defined by two strongly induced serine carboxypeptidase-like acyltransferases (*SCPL19/SNG2*) and several UGTs, together with *OPR4* and other oxidoreductases. The third set captured hormone-related stress regulation, with induced expression of *GH3.8*, *ACC oxidase*, *TIFY/JAZ* repressors, and *PIF1*, representing auxin-conjugating, ethylene-forming, and jasmonate-responsive genes. The fourth set displayed a pronounced membrane-associated and transcriptional stress-related signature, including *P4-type ATPase ALA*, very-long-chain enoyl-CoA reductase (*ECR*), and stress-responsive transcription factors (*HSF*,* NAC*,* WRKY*).

Taken together, the IDU and RWU classes revealed a multi-layered in vitro response profile, comprising detoxification, plastid-associated redox reactions, phenolic–oxylipin turnover, hormone-associated stress signalling, membrane-associated stress components, and stress-responsive TFs.

### Light-associated genes revealed divergent expression profiles in symbiosis

Although all protocorms grew in darkness, in vitro protocorms, both symbiotic and asymbiotic, were exposed to incidental, culture-related light. No visible greening was observed; however, transcriptomic analyses revealed divergent expression of light-associated genes, within the RSD and RWD classes, against a background of broadly repressed photosynthesis-related gene expression (Supplementary Table S6).

Light-associated regulatory components assigned to the RSD class included *HY5*, multiple UV-responsive genes, *PAL*, the HCF244–OHP assembly factors, and the phytochrome-associated SCF adaptor (*PCH-like*). This class also included genes associated with genome protection, such as *EHD*, *POT1a*, and *PHR1*, as well as stress-associated protein quality control, including molecular chaperones such as *Hsp70*/*Hsp90.* RWD-assigned genes included plastid-associated components such as *PSBO*/*OEC33*, *LHCa1*, *LHCa3*, and lycopene ε-cyclase (*LCY-e*) (Fig. 3; Table 1, Supplementary Tables S4, S5).

Together, these patterns indicate that light-associated genes were generally repressed in symbiotic protocorms relative to the asymbiotic control, while the strength of this repression differed between field- and in vitro-derived symbiotic responses.

## Discussion

### The in vitro system reproduced the major transcriptional features of orchid symbiosis

The separation of asymbiotic and in vitro symbiotic protocorms resembled transcriptional profiles reported for other orchid species [20, 61–63]. Enrichment analysis confirmed the induction of functions associated with symbiotic signalling, global transcriptional regulation and secondary metabolite synthesis [39, 64, 65]. The enriched expression of transporter genes, predominantly carrier-mediated, and genes linked to trehalose metabolism [16, 20, 61–64] was consistent with the proposed role of fungal carbon transfer during initial mycoheterotrophy [16, 20, 42, 61–64, 66, 67]. Upregulation of proteinases, protein-modifying enzymes, and homeostasis-related functions was in line with active protein quality control in protocorm symbiosis [16, 20]. Induction of *GA2ox*, a phytohormone-related gene, was consistent with its reported role in facilitating fungal colonisation during orchid seed germination and early protocorm development [24, 68]. The in vitro symbiotic transcriptome also showed induction of genes encoding signalling peptides described as regulators of arbuscular mycorrhizal symbiosis [69] and wall-associated signalling components, including WAK/WAK-like receptors. This pointed to enhanced wall-associated signalling during fungal colonisation [70].

### Physiological response to symbiosis is conserved but environmentally modulated

Having confirmed that our in vitro system reproduced the major transcriptional features of orchid protocorm symbiosis, we used field-derived protocorms as a natural context for evaluating how this programme was expressed outside controlled culture conditions. This comparison showed that, despite the lower ecological complexity of the in vitro system, protocorm responses to symbiosis were largely conserved: 94.2% of symbiosis-responsive genes in the field response (FS/IVA) showed the same regulatory direction (either upregulation or downregulation) as the in vitro symbiotic response (IVS/IVA), revealing a broad, conserved symbiotic blueprint across growth contexts [61–63]. This conservation extended to a broad gene set (Fig. 3; Supplementary Table S3) suggesting that mycoheterotrophy during the protocorm stage is underpinned by a conserved regulatory network. Yet environmental modulation was also present: expression differed for 2,485 genes, of which 1,290 showed opposing regulation across conditions, i.e. upregulation in the field response but downregulation in the symbiotic in vitro response, or vice versa (IUD and IDU classes, respectively). This inverted response pattern highlighted quantitative and qualitative differences between the field and in vitro symbiotic responses, likely reflecting fine-tuning of shared pathways to the environmental contexts.

Because the final RNA-seq dataset was not fully population-balanced, population-level variation cannot be formally excluded as a minor contributor to some FS–IVS differences. Nevertheless, the broad and functionally coherent nature of the observed patterns suggests that they mainly reflect modulation of the symbiotic transcriptional response by the contrasting field and in vitro contexts.

### Environmental context reshapes the conserved symbiotic programme

The conserved symbiotic transcriptional pattern described above was differentially shaped by field and in vitro contexts. The most prominent differences concerned nutrient transport, regulation of the plant–fungus interface, and stress-related signalling - three processes that are functionally linked during orchid protocorm symbiotic development.

#### Nutrient transport and regulation of the plant–fungus interface

In addition to common activation of nutrient-transport genes typical of orchid symbiosis [20, 61, 63], field-grown protocorms upregulated an additional set of transporters (IUD class) associated with sugar, amino-acid, phosphate, ion, and water movement. This broader transport signature may be connected with the greater ecological complexity of field conditions compared to in vitro cultures, including multiple *Tulasnella* strains detected in our samples (see Supplementary Table S2), associated soil microbes, and potential mycorrhizal links to adult orchids [34, 71–73]. Such complexity may broaden the range of possible carbon-, nutrient- and water-transfer routes and may also increase the need for dynamic regulation of the plant–fungus interface [16, 74, 75].

This interpretation is supported by field-associated transcriptional signatures of interface and cell-wall-integrity (CWI) regulation, including WAK-family wall kinases, consistent with their roles in wall-stress perception [76, 77] and mycorrhizal accommodation [70]. The CWI system integrates apoplast-associated signalling and cell-wall tension with local growth and immune modulation [69, 78, 79]. Markedly stronger upregulation of its components, together with membrane-wall integrity regulators including *CrRLK1L*, *COBRA*, and neutral ceramidase [80–82], in the IUD class of field protocorms, pointed to enhanced monitoring and adjustment of the interface, which may be particularly relevant under natural conditions. The physical constraints of the soil matrix and a more complex fungal context may increase the need to coordinate wall-stress perception at the cell surface with intracellular fungal accommodation and host cellular integrity. In contrast, the simplified in vitro system, based on a single compatible fungal isolate and agar medium, may attenuate the need for such extensive interface regulation and mechanosensory adjustment.

#### Stress-related regulation

Field and in vitro symbiotic protocorms also differed in the regulation of redox- and defence-associated pathways. While activation of glutathione transferases, phenolic metabolism, and ABC transporters represents a common feature of symbiotic establishment [63, 83, 84], field protocorms maintained expression of these genes at lower and more tightly coordinated levels. This pattern points to selective modulation rather than broad stress-like activation. Together with the coordinated activation of transcription factors linking hormonal, redox, and developmental signalling, this suggests that defence-related regulation in field protocorms remained integrated with growth and metabolism. Such organisation may buffer responses to fungal colonisation and soil microbiota, resulting in a lower stress signature than that observed under in vitro symbiotic conditions. Although data from mature roots cannot be directly extrapolated to protocorms, reduced expression of defence-related genes reported in roots of *Oeceoclades maculata* collected in nature [15] suggests that compatible orchid mycorrhizal interactions can be associated with limited, rather than excessive, defence activation.

In contrast, the in vitro response encompassed a broader and less integrated spectrum of stress-associated modules, including plastid-related redox processes, phenolic–oxylipin turnover, hormone-linked stress signalling, and stress-responsive transcription factors. This broader stress-associated transcriptional activation in vitro may be reinforced by culture conditions, including altered gas composition and ethylene accumulation [85], which may amplify redox- and defence-related signalling. Enhanced phenolic metabolism is frequently associated with protocorm browning in orchid in vitro cultures, a stress-related syndrome linked to reduced protocorm viability and survival [86]. Because brown or yellowing protocorms were not sampled, the transcriptional signal observed here may reflect an earlier molecular stage of stress-associated phenolic metabolism that precedes visible browning.

The stronger stress-related signature of IVS relative to FS protocorms therefore appears to reflect, at least in part, the artificial context of in vitro culture. However, comparison with IVA indicates that this response cannot be attributed to culture conditions alone. Rather, fungal colonisation appears to have modified how protocorms responded to incidental light exposure associated with culture inspection.

In asymbiotic protocorms, the induction of light-associated regulatory and photoprotective components, including elements of photosystem assembly and carotenoid biosynthesis (Fig. 3; Table 1, Supplementary Table S5) suggests that immature plastids remained responsive to even brief culture-related illumination. Such a response is consistent with early stages of plastid differentiation prior to photosynthetic competence [6, 87], but in protocorms normally developing underground, brief light exposure may represent a developmentally mismatched cue that imposes oxidative pressure on plastids not yet equipped for photosynthetic activity [88]. The ecological reference provided by soil-grown protocorms supports this interpretation, as they maintain a dark-grown plastid state analogous to skotomorphogenesis [89].

Although IVS showed a broader stress-related response than field protocorms, the light-associated plastid/redox component did not follow this general trend, suggesting that this part of the in vitro response was specifically modulated by symbiotic status. Although fungal partners are known to modulate host stress-related pathways [90, 91], this pattern may have a simpler explanation. Improved nutritional and metabolic status of symbiotic protocorms may reduce the need to engage plastid-related pathways associated with autonomous nutrition. This interpretation is ecologically plausible as light can penetrate a few millimetres into soil [92, 93], yet symbiotically supported protocorms of terrestrial orchids may avoid activating a potentially stressful plastid/redox response to such transient illumination.

Collectively, these findings indicate that field-grown protocorms maintain stress-related responses within a naturally integrated regulatory context, whereas in vitro culture introduces additional stress-associated cues. Consequently, in vitro conditions reproduce key molecular aspects of orchid symbiosis but do not fully capture the regulatory environment governing protocorm development under natural conditions.

## Conclusions

Our findings indicate that fungal colonisation induces a basal symbiotic transcriptional programme. The differences lie in how this core programme is shaped by environmental context. While in vitro systems are valuable for dissecting orchid –fungus interactions, their removal from the natural holobiont framework amplifies stress-associated responses relative to the adaptive regulation observed in field-grown protocorms.

Despite technical and logistical challenges, omics analyses of field-collected material yield coherent, biologically interpretable patterns that enable meaningful comparisons between natural and experimental systems and highlight symbiosis as a dynamic, ecosystem-embedded dialogue. Building on these results, future studies could use microcosm experiments or related approaches to separate individual environmental drivers and disentangle the relative contributions of fungal identity, soil structure, associated microbiota and abiotic conditions to protocorm transcriptional responses.

## Supplementary Information

Supplementary Material 1: D. majalis protocorm samples analysed in this study, indicating provenance, growth environment (field or in vitro); 2019 and 2021 refer to sampling years; mycorrhizal condition (symbiotic or asymbiotic), number of protocorms used for RNA isolation and their growth stage according Tesitelova et al.(2012); fungal isolate used for in vitro symbiotic culture (IVS); and fungal identity as revealed by MiCoP-based RNAseq data (see Table S2 for details). Supplementary Table S2: Fungal taxa detected in individual symbiotic protocorms based on MiCoP analysis.

Supplementary Material 2: Supplementary Table S3. Differential gene expression and functional enrichment results for pairwise contrasts between field symbiotic, in vitro symbiotic, and in vitro asymbiotic protocorms of D. majalis.

Supplementary Material 3: Supplementary Table S4. Functional enrichment of genes grouped by expression classes in D. majalis protocorms.

Supplementary Material 4: Supplementary Table S5. Transcripts underlying enriched functional categories across differential expression classes in D. majalis protocorms.

Supplementary Material 5: Supplementary Table S6 Fraction of photosynthetic genes that are not expressed or poorly expressed in the protocorms of D. majalis.

Supplementary Material 6: Supplementary Fig. S1. Field protocorm collection pipeline. Schematic overview of the procedure used to recover orchid protocorms germinating under natural field conditions from soil-buried seed packets. The protocorm photograph is illustrative and shows field-recovered Neottia ovata protocorms, not the Dactylorhiza majalis material used for RNA-seq. Supplementary Fig. S2. Dactylorhiza majalis protocorms from the Psary population grown in vitro. A, Asymbiotic culture on ¼-2 medium. B, Symbiotic culture on oat meal agar (OMA) medium with Tulasnella irregularis isolate. Black scale bars represent 1 cm. Arrows indicate examples of protocorms selected for RNA-seq analysis.

## Data Availability

The datasets (The reads and the raw count matrices) generated and/or analysed during the current study are available in the NCBI database https://www.ncbi.nlm.nih.gov/geo/query/acc.cgi ; GEO accession GSE315930.

## Abbreviations

FS: field grown symbiotic protocorms
IVS: in vitro grown symbiotic protocorms
IVA: in vitro grown asymbiotic protocorms
RSU: relative stronger upregulation
RWU: relative weaker upregulation
RSD: relative stronger downregulation
RWD: relative weaker downregulation
IUD: inverted: Up in field response / Down in in vitro response (upregulated in FS/IVA and downregulated in IVS/IVA
IDU: inverted: Down in field response / Up in in vitro response(downregulated in FS/IVA and upregulated in IVS/IVA)

## References

[1] Dearnaley JDW, Martos F, Selosse MA. Orchid mycorrhizas: molecular ecologyy, physiology, evolution and conservation aspects. In: Hock B, editor. Fungal Associations. 2nd ed. Berlin–Heidelberg: Springer; 2012. pp. 207–30. 10.1007/978-3-642-30826-0_12.

[2] Selosse MA, Petrolli R, Mujica MI, Laurent L, Perez-Lamarque B, Figura T, Bourceret A, Jacquemyn H, Li T, Gao J, et al. The Waiting Room Hypothesis revisited by orchids: were orchid mycorrhizal fungi recruited among root endophytes? Ann Bot. 2022. 10.1093/aob/mcab134. PMC8835631 34718377

[3] Selosse MA, Alaux PL, Deloche L, Delannoy E, Minasiewicz J, Tsiftsis S, Figura T, Martos F. Mixotrophy in orchids: facts, questions, and perspectives. New Phytol. 2025. 10.1111/nph.70106. PMC12059541 40195594

[4] Rasmussen HN. Terrestrial orchids: From seed to mycotrophic plant. Cambridge, UK: Cambridge University Press; 1995.

[5] Dearnaley J, Perotto S, Selosse M-A. Structure and development of orchid mycorrhizas.In: Martin F ,editor. Molecular Mycorrhizal Symbiosis. John Wiley & Sons, Ltd, Chichester, 2016. p. 63–86. 10.1002/9781118951446.ch5.

[6] Yeung EC. A perspective on orchid seed and protocorm development. Bot Stud. 2017. 10.1186/s40529-017-0188-4. PMC5544657 28779349

[7] Peterson RL, Massicotte HB. Exploring structural definitions of mycorrhizas, with emphasis on nutrient-exchange interfaces. Can J Bot. 2004. 10.1139/b04-071.

[8] Kuga Y, Sakamoto N, Yurimoto H. Stable isotope cellular imaging reveals that both live and degenerating fungal pelotons transfer carbon and nitrogen to orchid protocorms. New Phytol. 2014. 10.1111/nph.12700. 24494717

[9] Leake JR. Tansley Review 69. The biology of myco-heterotrophic (‘saprophytic’) plants. New Phytol. 1994. 10.1111/j.1469-8137.1994.tb04272.x. 33874520

[10] Merckx V, editor. Mycoheterotrophy: The biology of plants living on fungi. New York; Springer, 2013 . 10.1007/978-1-4614-5209-6.

[11] Rasmussen HN, Rasmussen FN. Orchid mycorrhiza: implications of a mycophagous lifestyle. Oikos. 2009. 10.1111/j.1600-0706.2008.17116.x.

[12] Selosse MA, Roy M. Green plants that feed on fungi: facts and questions about mixotrophy. Trends Plant Sci. 2009. 10.1016/j.tplants.2008.11.004. 19162524

[13] Dearnaley JDW. Further advances in orchid mycorrhizal research. Mycorrhiza. 2007. 10.1007/s00572-007-0138-1. 17582535

[14] Genre A, Lanfranco L, Perotto S, Bonfante P. Unique and common traits in mycorrhizal symbioses. Nat Rev Microbiol. 2020. 10.1038/s41579-020-0402-3. 32694620

[15] Valadares RBS, Perotto S, Lucheta AR, Santos EC, Oliveira RM, Lambais MR. Proteomic and transcriptomic analyses indicate metabolic changes and reduced defense responses in mycorrhizal roots of *Oeceoclades maculata* (*Orchidaceae*) collected in nature. J Fungi. 2020. 10.3390/jof6030148. PMC7558880 32858792

[16] Fochi V, Chitarra W, Kohler A, Voyron S, Singan VR, Lindquist EA, Barry KW, Girlanda M, Grigoriev IV, Martin F, et al. Fungal and plant gene expression in the *Tulasnella calospora–Serapias vomeracea* symbiosis provides clues about nitrogen pathways in orchid mycorrhizas. New Phytol. 2017. 10.1111/nph.14279. 27859287

[17] Jąkalski M, Minasiewicz J, Caius J, May M, Selosse M-A, Delannoy E. The genomic impact of mycoheterotrophy in orchids. Front Plant Sci. 2021. 10.3389/fpls.2021.632033. PMC8220222 34177974

[18] Wei Y, Li J, Jin J, Gao J, Xie Q, Lu C, Zhu G, Yang F. Centenary progress on Orchidaceae research: a bibliometric analysis. Genes. 2025. 10.3390/genes16030336. PMC11942143 40149487

[19] Bidartondo MI. The evolutionary ecology of myco-heterotrophy. New Phytol. 2005. 10.1111/j.1469-8137.2005.01429.x. 15998389

[20] Perotto S, Rodda M, Benetti A, Sillo F, Ercole E, Rodda M, Girlanda M, Murat C, Balestrini R. Gene expression in mycorrhizal orchid protocorms suggests a friendly plant-fungus relationship. Planta. 2014. 10.1007/s00425-014-2062-x. 24760407

[21] Dearnaley JDW, Cameron DD. Nitrogen transport in the orchid mycorrhizal symbiosis – further evidence for a mutualistic association. New Phytol. 2017. 10.1111/nph.14357. 27891646

[22] Miura C, Yamaguchi K, Miyahara R, Yamamoto T, Fuji M, Yagame T, Imaizumi-Anraku H, Yamato M, Shigenobu S, Kaminaka H. The mycoheterotrophic symbiosis between orchids and mycorrhizal fungi possesses major components shared with mutualistic plant-mycorrhizal symbioses. Mol Plant-Microbe Int. 2018. 10.1094/MPMI-01-18-0029-R. 29649962

[23] Zhao Z, Wang Y, Yang L, Qian X, Yang A, Liu J, Jacquemyn H, Li T, Xing X. Metabolic shifts and nutrient transfer patterns in orchid seeds during symbiotic germination. Plant Cell Environ. 2025. 10.1111/pce.15612. 40369997

[24] Chen, Li Y, Yin Y, Li J, Li L, Wu K, Fang L, Zeng S. Gibberellic acid inhibits *Dendrobium nobile*—*Piriformospora* symbiosis by regulating the expression of cell wall metabolism genes. Biomolecules. 2023. 10.3390/biom13111649. PMC10669577 38002331

[25] Miura C, Tominaga T, Kaminaka H. Different roles of the phytohormone gibberellin in the wide-spread arbuscular mycorrhiza and in orchid mycorrhiza. Curr Opin Plant Biol. 2024b. 10.1016/j.pbi.2024.102627. 39250880

[26] Steidinger BS. Mycorrhizal arbitrage, a hypothesis: How mycoheterotrophs could profit from inefficiencies in the biological marketplace. Funct Ecol. 2024. 10.1111/1365-2435.14609.

[27] Cameron DD, Johnson I, Read DJ, Leake JR. Giving and receiving: measuring the carbon cost of mycorrhizas in the green orchid, *Goodyera repens*. New Phytol. 2008. 10.1111/j.1469-8137.2008.02533.x. 18627489

[28] Kauth PJ, Dutra D, Johnson TR, Stewart SL, Kane ME, Vendrame WA. 2008 Techniques and applications of *in vitro* orchid seed germination. In: Teixeira da Silva, JA, editor. Floriculture, Ornamental and Plant Biotechnology: Advances and Topical Issues, Vol. 5. Global Science Books, Isleworth, UK; 2008. pp. 375–391. https://www.humboldtorchids.org/Orchidseedbookchapter.pdf.

[29] Masuhara G, Katsuya K. *In situ* and *in vitro* specificity between Rhizoctonia spp. and Spiranthes sinensis (Persoon) Ames, var. amoena (M. Bieberstein) Hara (Orchidaceae). New Phytol. 1994. . 10.1111/j.1469-8137.1994.tb02974.x 33874389

[30] Matarese G, La Cava A, Horvath TL. *In vivo* veritas, *in vitro* artificia. Trends Mol Med. 2012; 10.1016/j.molmed.2012.05.003. PMC3477516 22682514

[31] Vinogradova TN, Andronova EV. Development: Development of Orchid Seeds and Seedlings. In: Arditti J, editor. Orchid Biology: Reviews and Perspectives, VIII. Dordrecht: Springer; 2002. pp. 167–234.

[32] Harzli I, Kömpe YÖ. Assessing the effectiveness of *in vitro* and *in situ* symbiotic seed germination: case of *Anacamptis papilionacea* (L) R.M. Bateman, Pridgeon M W Chase. Symbiosis. 2023. 10.1007/s13199-023-00950-8.

[33] Rasmussen HN, Dixon KW, Jersáková J, Těšitelová T. Germination and seedling establishment in orchids: a complex of requirements. Ann Bot. 2015. 10.1093/aob/mcv087. PMC4549959 26271118

[34] Read DJ, Haggar J, Magkourilou E, Durant E, Johnson D, Leake JR, Field KJ. Photosynthate transfer from an autotrophic orchid to conspecific heterotrophic protocorms through a common mycorrhizal network. New Phytol. 2024. 10.1111/nph.19810. 38757767

[35] Calevo J, Alibrandi P, Voyron S, Girlanda M, Peroto S. Breaking boundaries: fungi in the rhizoctonia species complex exhibit systemic colonization in three terrestrial orchid species. bioRxiv. 2025. 10.1101/2025.01.08.631981.

[36] Ventre-Lespiaucq A, Jacquemyn H, Rasmussen HN, Méndez M. Temporal turnover in mycorrhizal interactions: a proof of concept with orchids. New Phytol. 2021. 10.1111/nph.17291. 33621346

[37] Tedersoo L, Bahram M, Zobel M. How mycorrhizal associations drive plant population and community biology. Science. 2020. 10.1126/science.aba1223. 32079744

[38] Pujasatria GC, Miura C, Yamaguchi K, Shigenobu S, Kaminaka H. Colonization by orchid mycorrhizal fungi primes induced systemic resistance against necrotrophic pathogen. Front Plant Sci. 2024;2024. 10.3389/fpls.2024.1447050. PMC11322130 39145195

[39] Zhao X, Zhang J, Chen C, Yang J, Zhu H, Liu M, Lv F. Deep sequencing-based comparative transcriptional profiles of *Cymbidium hybridum* roots in response to mycorrhizal and non-mycorrhizal beneficial fungi. BMC Genomics. 2014. 10.1186/1471-2164-15-747. PMC4162972 25174959

[40] Jacquemyn H, Deja A, De hert K, Cachapa Bailarote B, Lievens B. Variation in mycorrhizal associations with Tulasnelloid fungi among populations of five *Dactylorhiza* species. PLoS ONE. 2012. 10.1371/journal.pone.0042212. PMC3411701 22870305

[41] Figura T, Tylová E, Jersáková J, Vohník M, Ponert J. Fungal symbionts may modulate nitrate inhibitory effect on orchid seed germination. Mycorrhiza. 2021. 10.1007/s00572-021-01021-w. 33492496

[42] Ponert J, Šoch J, Vosolsob S, Čiháková K, Lipavská H. Integrative study supports the role of trehalose in Carbon Transfer from fungi to mycotrophic orchid. Front Plant Sci. 2021. 10.3389/fpls.2021.793876. PMC8695678 34956293

[43] Chase MW, Cameron KM, Freudenstein JV, Pridgeon AM, Salazar G, den Berg C, Schuiteman A. An updated classification of *Orchidaceae*. Bot J Lin Soc. 2015. 10.1111/boj.12234.

[44] Jacquemyn H, Waud M, Merckx VSFT, Brys R, Tyteca D, Hedrén M, Lievens B. Habitat-driven variation in mycorrhizal communities in the terrestrial orchid genus *Dactylorhiza*. Sci Rep. 2016. 10.1038/srep37182. PMC5121631 27883008

[45] Brundrett MC, Scade A, Batty AL, Dixon KW, Sivasithamparam K. Development of *in situ* and *ex situ* seed baiting techniques to detect mycorrhizal fungi from terrestrial orchid habitats. Mycol Res. 2003. 10.1017/S0953756203008463. 14635769

[46] Tešitelová T, Tešitel J, Jersáková J, Ríhová G, Selosse M-A. Symbiotic germination capability of four Epipactis species (Orchidaceae) is broader than expected from adult ecology. Am J Bot. 2012. 10.3732/ajb.1100503. 22688426

[47] Warcup JH, Talbot PHB. Perfect states of rhizoctonias associated with orchids. New Phytol. 1967. 10.1111/j.1469-8137.1967.tb05434.x.

[48] LaPierre N, Mangul S, Alser M, Mandric I, Wu NC, Koslicki D, Eskin E. MiCoP: microbial community profiling method for detecting viral and fungal organisms in metagenomic samples. BMC Genomics. 2019. 10.1186/s12864-018-5396-0. PMC6551237 31167634

[49] Ponert J, Figura T, Vosolsobě S, Lipavská H, Vohník M, Jersáková J. Asymbiotic germination of mature seeds and protocorm development of *Pseudorchis albida* (Orchidaceae) are inhibited by nitrates even at extremely low concentrations. Botany. 2013. 10.1139/cjb-2013-0082.

[50] Bolger AM, Lohse M, Usadel B. Trimmomatic: a flexible trimmer for Illumina sequence data. Bioinformatics. 2014. 10.1093/bioinformatics/btu170. PMC4103590 24695404

[51] Bushnell B. BBMap: a fast, accurate, splice-aware aligner. Lawrence Berkeley National Laboratory (LBNL), Berkeley, CA. 2014; https://escholarship.org/uc/item/1h3515gn

[52] R Core Team. R Core Team 2023 R: A language and environment for statistical computing. R foundation for statistical computing. R Foundation for Statistical Computing; 2023. https://www.R-project.org/.

[53] Lambert I, Paysant-Le Roux C, Colella S, Martin-Magniette M-L. DiCoExpress: a tool to process multifactorial RNAseq experiments from quality controls to co-expression analysis through differential analysis based on contrasts inside GLM models. Plant Methods. 2020. 10.1186/s13007-020-00611-7. PMC7216733 32426025

[54] Baudry K, Roux CP, Le, Colella S, Castandet B, Martin ML. Analyzing Multifactorial RNA-Seq Experiments with DicoExpress. J Vis Exp. 2022. 10.3791/62566. 35969075

[55] Robinson MD, McCarthy DJ, Smyth GK. edgeR: A Bioconductor package for differential expression analysis of digital gene expression data. Bioinformatics. 2009. 10.1093/bioinformatics/btp616. PMC2796818 19910308

[56] McCarthy DJ, Chen Y, Smyth GK. Differential expression analysis of multifactor RNA-Seq experiments with respect to biological variation. Nucleic Acids Res. 2012. 10.1093/nar/gks042. PMC3378882 22287627

[57] Benjamini Y, Hochberg Y. 1995 Controlling the false discovery rate - A practical and powerful approach to multiple testing. J Royal Statist Soc, Series B. 1995; 10.1111/j.2517-6161.1995.tb02031.x.

[58] Rigaill G, Balzergue S, Brunaud V, Blondet E, Rau A, Rogier O, Caius J, Maugis-Rabusseau C, Soubigou-Taconnat L, Aubourg S, et al. Synthetic data sets for the identification of key ingredients for RNA-seq differential analysis. Brief Bioinform. 2018. 10.1093/bib/bbw092. 27742662

[59] Van Bel M, Proost S, Van Neste C, Deforce D, Van de Peer Y, Vandepoele K. TRAPID: An efficient online tool for the functional and comparative analysis of de novo RNA-Seq transcriptomes. Genome Biol. 2013. 10.1186/gb-2013-14-12-r134. PMC4053847 24330842

[60] Schwacke R, Ponce-Soto GY, Krause K, Bolger AM, Arsova B, Hallab A, Gruden K, Stitt M, Bolger ME, Usadel B. MapMan4: A refined protein classification and annotation framework applicable to multi-omics data analysis. Mol Plant. 2019. 10.1016/j.molp.2019.01.003 30639314

[61] Balestrini R, Nerva L, Sillo F, Girlanda M, Perotto S. Plant and fungal gene expression in mycorrhizal protocorms of the orchid *Serapias vomeracea* colonized by *Tulasnella calospora*. Plant Signal Behav. 2014. 10.4161/15592324.2014.977707. PMC4623096 25482758

[62] Chen J, Liu SS, Kohler A, Yan B, Luo HM, Chen XM, Guo SX. iTRAQ and RNA-seq analyses provide new insights into regulation mechanism of symbiotic germination of *Dendrobium officinale* seeds (*Orchidaceae*). J Proteome Res. 2017. 10.1021/acs.jproteome.6b00999 . 28488443

[63] Chen J, Tang Y, Kohler A, Lebreton A, Xing Y, Zhou D, Li Y, Martin FM, Guo S. Comparative transcriptomics analysis of the symbiotic germination of *D. officinale* (*Orchidaceae)* with emphasis on plant cell wall modification and cell wall-degrading enzymes. Front Plant Sci. 2022. 10.3389/fpls.2022.880600. PMC9120867 35599894

[64] Jin Y, Liu H, Luo D, Yu N, Dong W, Wang C, Zhang X, Dai H, Yang J, Wang E. DELLA proteins are common components of symbiotic rhizobial and mycorrhizal signalling pathways. Nat Commun. 2016. 10.1038/ncomms12433. PMC4990646 27514472

[65] Jiang Y, Hu X, Yuan Y, Guo X, Chase MW, Ge S, Li J, Fu J, Li K, Hao M, et al. The *Gastrodia menghaiensis* (*Orchidaceae*) genome provides new insights of orchid mycorrhizal interactions. BMC Plant Biol. 2022. 10.1186/s12870-022-03573-1 . PMC8988336 35392808

[66] Li MH, Liu KW, Li Z, et al. Genomes of leafy and leafless *Platanthera* orchids illuminate the evolution of mycoheterotrophy. Nat Plants. 2022. 10.1038/s41477-022-01127-9. PMC9023349 35449401

[67] Zhao DK, Mou ZM, Ruan YL. Orchids acquire fungal carbon for seed germination: pathways and players. Trends Plant Sci. 2024. 10.1016/j.tplants.2024.02.001. 38423891

[68] Miura C, Furui Y, Yamamoto T, Kanno Y, Honjo M, Yamaguchi K, Suetsugu K, Yagame T, Seo M, Shigenobu S, et al. Autoactivation of mycorrhizal symbiosis signaling through gibberellin deactivation in orchid seed germination. Plant Physiol. 2024a. 10.1093/plphys/kiad517. PMC10756758 37776523

[69] Mamaeva A, Makeeva A, Ganaeva D. The small key to the treasure chest: endogenous plant peptides involved in symbiotic interactions. Plants. 2025. 10.3390/plants14030378. PMC11820598 39942939

[70] Zhang X, Jia S, He Y, Wen J, Li D, Yang W, Yue Y, Li H, Cheng K, Zhang X. Wall-associated kinase GhWAK13 mediates arbuscular mycorrhizal symbiosis and *Verticillium* wilt resistance in cotton. New Phytol. 2024. 10.1111/nph.19468. 38095050

[71] Diez JM. Hierarchical patterns of symbiotic orchid germination linked to adult proximity and environmental gradients. J Ecol. 2007. 10.1111/j.1365-2745.2006.01194.x.

[72] McCormick MK, Lee Taylor D, Juhaszova K, Burnett RK, Whigham DF, O’Neill JP. Limitations on orchid recruitment: Not a simple picture. Mol Ecol. 2012. 10.1111/j.1365-294X.2012.05468.x. 22272942

[73] Calevo J, Duffy KJ. Interactions among mycorrhizal fungi enhance the early development of a Mediterranean orchid. Mycorrhiza. 2023. 10.1007/s00572-023-01118-4. PMC10442268 37436449

[74] Peterson RL, Uetake Y, Bonfante P, Faccio A. The interface between fungal hyphae and orchid protocorm cells. Can J Bot. 1996. 10.1139/b96-223.

[75] Rasmussen HN, Whigham DF. Phenology of roots and mycorrhiza in orchid species differing in phototrophic strategy. New Phytol. 2002. 10.1046/j.1469-8137.2002.00422.x. 33873451

[76] Decreux A, Messiaen J. Wall-associated kinase WAK1 interacts with cell wall pectins in a calcium-induced conformation. Plant Cell Physiol. 2005. 10.1093/pcp/pci026. 15769808

[77] Kohorn BD. Cell wall-associated kinases and pectin perception. J Exp Bot. 2016. 10.1093/jxb/erv467. 26507892

[78] Ren H, Gray WM. SAUR Proteins as effectors of hormonal and environmental signals in plant growth. Mol Plant. 2015. 10.1016/j.molp.2015.05.003. PMC5124491 25983207

[79] Cheung AY. FERONIA: a receptor kinase at the core of a global signaling network. Annu Rev Plant Biol. 2024. 10.1146/annurev-arplant-102820-103424. PMC12034098 38424067

[80] Ko J-H, Kim JH, Jayanty SS, Howe GA, Han K-H. Loss of function of COBRA, a determinant of oriented cell expansion, invokes cellular defence responses in *Arabidopsis thaliana*. J Exp Bot. 2006. 10.1093/jxb/erl052. 16873454

[81] Baez LA, Tichá T, Hamann T. Cell wall integrity regulation across plant species. Plant Mol Biol. 2022. 10.1007/s11103-022-01284-7. PMC9213367 35674976

[82] Zienkiewicz A, Gömann J, König S, Herrfurth C, Liu YT, Meldau D, Feussner I. Disruption of *Arabidopsis* neutral ceramidases 1 and 2 results in specific sphingolipid imbalances triggering different phytohormone-dependent plant cell death programmes. New Phytol. 2020. 10.1111/nph.16336 . 31758808

[83] Balestrini R, Gómez-Ariza J, Lanfranco L, Bonfante P. Laser microdissection reveals that transcripts for five plant and one fungal phosphate transporter genes are contemporaneously present in arbusculated cells. Mol Plant-Microbe Interact. 2007. 10.1094/MPMI-20-9-1055. 17849708

[84] Valadares RBS, Perotto S, Santos EC, Lambais MR. Proteome changes in *Oncidium sphacelatum* (*Orchidaceae*) at different trophic stages of symbiotic germination. Mycorrhiza. 2014. 10.1007/s00572-013-0547-2. 24310930

[85] Jackson MB, Abbott AJ, Belcher AR, Hall KC, Butler R, Cameron J. Ventilation in plant tissue cultures and effects of poor aeration on ethylene and carbon dioxide accumulation, oxygen depletion and explant development. Ann Bot. 1991. 10.1093/oxfordjournals.aob.a088127.

[86] Adamo M, Chialva M, Calevo J, Rose S, De, Girlanda M, Perotto S, Balestrini R. The dark side of orchid symbiosis: Can *Tulasnella calospora* decompose host tissues? Int J Mol Sci. 2020. 10.3390/ijms21093139. PMC7247694 32365577

[87] Yeung EC. The orchid embryo -an embryonic protocorm. Botany. 2022. 10.1139/cjb-2022-001.

[88] op den Camp RGL, Przybyla D, Ochsenbein C, Laloi C, Kim C, Danon A, Wagner D, Hideg E, Göbel C, Feussner I, et al. Rapid induction of distinct stress responses after the release of singlet oxygen in *Arabidopsis*. Plant Cell. 2003. 10.1105/tpc.014662. PMC197298 14508004

[89] Seluzicki A, Burko Y, Chory J. Dancing in the dark: darkness as a signal in plants. Plant Cell Environ. 2017. 10.1111/pce.12900. PMC6110299 28044340

[90] Aroca R, Porcel R, Ruiz-Lozano JM. How does arbuscular mycorrhizal symbiosis regulate root hydraulic properties and plasma membrane aquaporins in Phaseolus vulgaris under drought, cold or salinity stresses? New Phytol. 2007. 10.1111/j.1469-8137.2006.01961.x. 17286829

[91] Volpin H, Elkind Y, Okon Y, Kapulnik Y. A vesicular arbuscular mycorrhizal fungus (*Glomus intraradix*) induces a defense response in Alfalfa roots. Plant Physiol. 1994. 10.1104/pp.104.2.683. PMC159247 12232119

[92] Woolley JT, Stoller EW. Light penetration and light-induced seed germination in soil. Plant Physiol. 1978. 10.1104/pp.61.4.597. PMC1091925 16660344

[93] Tester M, Morris C. The penetration of light through soil. Plant Cell Environ. 1987. 10.1111/j.1365-3040.1987.tb01607.x .

